# Supplementary material for: Enhanced Assessment of Cross-Reactive Antigenic Determinants within the Spike Protein
Source: Int J Mol Sci. 2024 Jul 26;25(15):8180. doi: 10.3390/ijms25158180 (PMC11311977; doi:10.3390/ijms25158180)
Supplement: Supplementary file 1 [file ijms-25-08180-s001.zip › ijms-3068142-supplementary.pdf]

– Supplementary Information –

Table S1. IgG epitopes mapped for Sars-CoV-2 Spike protein using a pool of sera from patients with COVID-19 disease and cross-reactivity against DENV serotypes 1-4 (Taxid: 12637).

Peptides	Cross-reactivity*	Specificity
FNDGVYFASTEKSNI	DENV2	Sars-CoV-2, BatCoV-RaTG13
DSKTQSLLIVNNATN	DENV3	Sars-CoV-2, BatCoV-RaTG13
LGVYYHKNNK	No	Sars-CoV-2, BatCoV-RaTG13
LMDLEGKQGNFKNLR	DENV3	Sars-CoV-2, BatCoV-RaTG13
NLVRDLPQGF	No	Sars-CoV-2, BatCoV-RaTG13
RSVLTPGDSSS	Gram-positive bacteria and rodent	Sars-CoV-2, BatCoV-RaTG13
GAAAYYVGYL	No	Sars-CoV-2, BatCoV-RaTG13, SARS-CoV-1, MERS-CoV
GIYQTSNFRV	DENV2	Sars-CoV-2, BatCoV-RaTG13, SARS-CoV-1
KRISNCVADYSVLYN	No	Sars-CoV-2, BatCoV-RaTG13, SARS-CoV-1
YADSFVIRGD	No	Sars-CoV-2, BatCoV-RaTG13, SARS-CoV-1
GKIADYNYKL	DENV1	Sars-CoV-2, BatCoV-RaTG13, SARS-CoV-1
LDSKVGGNYN	No	SARS-CoV-2
LKPFERDIST	No	Sars-CoV-2, BatCoV-RaTG13, SARS-CoV-1
PLQSYGFQPTGVGY	No	Sars-CoV-2, BatCoV-RaTG13, SARS-CoV-1
NKKFLPFQQF	DENV2	Sars-CoV-2, BatCoV-RaTG13, SARS-CoV-1
DTTDAVRDPQ	No	Sars-CoV-2, BatCoV-RaTG13, SARS-CoV-1
NQVAVLYQDV	No	Sars-CoV-2, BatCoV-RaTG13, SARS-CoV-1
ADQLTPTWRV	No	Sars-CoV-2, BatCoV-RaTG13, SARS-CoV-1
IGAEHVNNSY	DENV2	Sars-CoV-2, BatCoV-RaTG13, SARS-CoV-1
TQTNSPRRAR	DENV 1 and 3	Sars-CoV-2, BatCoV-RaTG13, SARS-CoV-1
SIIAYTMSL	No	Sars-CoV-2, BatCoV-RaTG13, SARS-CoV-1
AYSNNSIAIP	No	Sars-CoV-2, BatCoV-RaTG13, SARS-CoV-1
AVEGD	No	Sars-CoV-2, BatCoV-RaTG13
KQIYK	Several organisms and DENV2	Sars-CoV-2, BatCoV-RaTG13
DFGGF	Several organisms and DENV1	Sars-CoV-2, BatCoV-RaTG13, SARS-CoV-1, MERS-CoV
LPDPSKPSKRSFIED	DENV1 and 2	Sars-CoV-2, BatCoV-RaTG13, SARS-CoV-1, MERS-CoV
LPPLLTDEMI	DENV3	Sars-CoV-2, BatCoV-RaTG13, SARS-CoV-1, MERS-CoV, HCoV_OC43, HCoV_HKU1
ALLAGTITSGWTFGA	DENV1	Sars-CoV-2, BatCoV-RaTG13, SARS-CoV-1
QMAYRFNGIG	No	Sars-CoV-2, BatCoV-

		RaTG13, SARS-CoV-1, MERS-CoV
KLIANGFNSA	Fungi	Sars-CoV-2, BatCoV-RaTG13, SARS-CoV-1, MERS-CoV, HCoV_OC43, HCoV_HKU1, HCoV-NL63, HCoV-229E
VVNQNAQALN	DENV1	Sars-CoV-2, BatCoV-RaTG13, SARS-CoV-1, MERS-CoV, HCoV_OC43, HCoV_HKU1, HCoV-NL63, HCoV-229E
GAISSVLNDI	DENV1 and 3	Sars-CoV-2, BatCoV-RaTG13, SARS-CoV-1, MERS-CoV, HCoV_OC43, HCoV_HKU1
LITGRLQSLQ	No	Sars-CoV-2, BatCoV-RaTG13, SARS-CoV-1, HCoV-NL63, HCoV-229E
AEIRA	No	Sars-CoV-2, BatCoV-RaTG13, SARS-CoV-1
GYHLMSFPQS	No	Sars-CoV-2, BatCoV-RaTG13, SARS-CoV-1
REGVFVSNGTHW	DENV3	Sars-CoV-2, BatCoV-RaTG13, SARS-CoV-1
EPQII	Several organisms	Sars-CoV-2, BatCoV-RaTG13, SARS-CoV-1
TVYDPLQPEL	No	Sars-CoV-2, BatCoV-RaTG13, SARS-CoV-1
KEIDRLNEVAK	Fungi, DENV1 and 3	Sars-CoV-2, BatCoV-RaTG13, SARS-CoV-1
SLIDLQELGK	No	Sars-CoV-2, BatCoV-RaTG13, SARS-CoV-1
FDEDDSEPVI	No	Sars-CoV-2, BatCoV-RaTG13, SARS-CoV-1

*Cross-reactivity was performed using the Basic Local Alignment Search Tool (BLAST) and was considered if sequences matches at least four amino acids

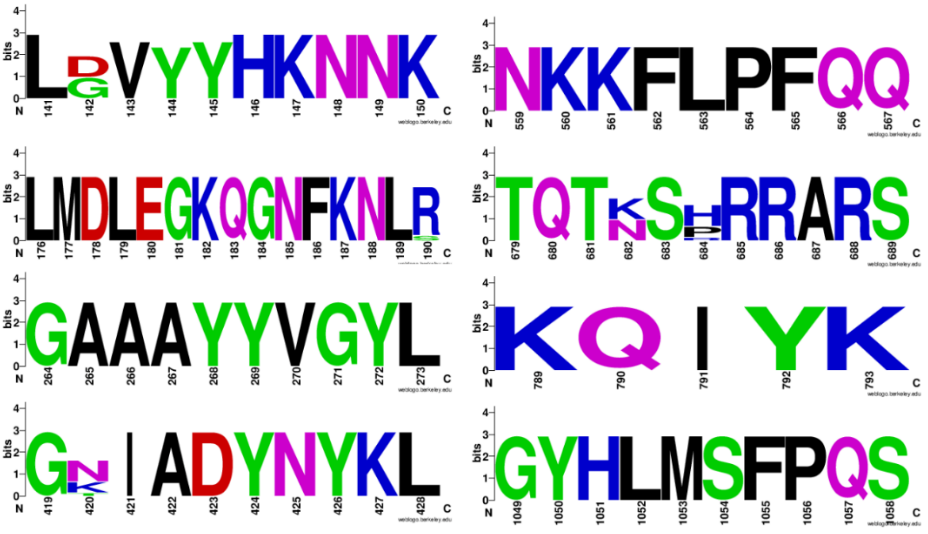

Figure S1. Sequence logos for spike protein cross-reactive DENV-identified sequences in Sars-CoV-2 variants of concern (Wuhan [wild type], Alpha, Beta, Delta, Gamma, Omicron BA.1, Omicron BA.2, Omicron BA.2.12.1, Omicron BA.4, Omicron BA.5) showing residues conservation. The selected amino acid sequence is shown on the X-axis. The height of each letter on the Y-axis indicates its relative frequency. N, N-terminal end; C, C-terminal end.

```
SARS-CoV-2     ----MFVFLVLLPLV-----------SSQCVNLT-----TRTQLPPAYTNSFTR----GVY-----------------------------------------YP-DKVFR 44
SARS           ----MFIFLLFLTLT-------------SGSDLDRCTTFDDVQAPNYTQHTSSMR---GVY-----------------------------------------YP-DEIFR 48
MERS-CoV       MIHSVFLLMFLLTPTESYVDVGPDSVKSACIEVDIQQTFFDKTWPR----PIDVSKADGII-----------------------------------------YPQGRTYS 65
HCoV OC43      ----MFLILLISLPT-AFAVIG----DLKCTSDNIND--KDTGPPPISTDTVDVTNGLGTY-----------------------------------------YVLDRVYL 58
HCoV NL63      --MKLFLILLVLPLASCFFTCNSNA-NLSMLQLGVPDNSSTIVTGLLPTHWFCANQSTSVYSANGFFYIDVGNHRSAFALHTGYYDANQYYIYVTNEIGL 97
HCoV HKU1      ----MFLIIFILPTT--LAVIG----DFNCTNSFIND--YNKTIPRISEDVVDVSLGLGTY-----------------------------------------YVLNRVYL 57
HCoV 229E      ----MFVLLVA---------------------------------------------------------------------------------YAL----- 10
BatCoV-RaTG13  ----MFVFLVLLPLV-----------SSQCVNLT-----TRTQLPPAYTNSSTR----GVY-----------------------------------------YP-DKVFR 44

SARS-CoV-2     SSV-----------------------------LHSTQDLFLPF----FSNVTWFHAIHVSGTNGTKR-------------------------------------- 78
SARS           SDT-----------------------------LYLTQDLFLPF----YSNVTGFHTINHT------------------------------------------ 75
MERS-CoV       NIT-----------------------------ITYQG---LFPYQG-DHGDMYVYSAGHATGTTPQKL-------------------------------------- 100
HCoV OC43      NTT-----------------------------LFLNG---YYPTSGSTYRNMALKGSVLLSRLWFKPP-------------------------------------- 94
HCoV NL63      NASVTLKICKFSRNTTFDFLSNASSSFDCIVNLLFTEQLGAPLGITISGETVRLHLYNVTRTFYVPAAYKLTKLSVKCYFNYSCVFSVVNATVTVNVTTH 197
HCoV HKU1      NTT-----------------------------LLFTG---YFPKSGANFRDLALKGSKYLSTLWYKPP-------------------------------------- 93
HCoV 229E      --------------------------------LHIAG---------------CQTTNGLNTSYS-------------------------------------- 27
BatCoV-RaTG13  SSV-----------------------------LHLTQDLFLPF----FSNVTWFHAIHVSGTNGIKR-------------------------------------- 78

SARS-CoV-2     --------FDNP----VLPFNDGVYFASTEKSNIIRG------------------WIFGTTLDSKT----------QSLLIVNN------------------ATNVV 127
SARS           --------FGNP----VIPFKDGIYFAATEKSNVVRG------------------WVFGSTMNNKS----------QSVIIINN------------------STNVV 124
MERS-CoV       --------FVANYSQDVKQFANGF-VVRIGAAANSTGTVIISPSTSATIRKIYPAFMLGSSVGNFSDGKMGRFFNHTLVLLP------------------DGCGT 178
HCoV OC43      --------FLSD-------FINGI-FAKVKNTKVIKDRVMYSE----------FPAITIGSTFVNTS-----------YSVVVQPRTINSTQDGDNKLQGLLE 160
HCoV NL63      NGRVVNYTVCDD----CNGYTDNI-FSVQQDGRIPNGFP--------------FNNWFL---LTNGS-----------TLV------------------DGVSR 250
HCoV HKU1      --------FLSD-------FNNGI-FSKVKNTKLYVNNTLYSE----------FSTIVIGSVFVNTS----------YTIVVQPH------------------NGILE 147
HCoV 229E      --------VCNG----CVGYSENV-FAVESGGYIPSDFA--------------FNNWFL---LTNTS-----------SVV------------------DGVVR 72
BatCoV-RaTG13  --------FDNP----VLPFNDGVYFASTEKSNIIRG------------------WIFGTTLDSKT----------QSLLIVNN------------------ATNVV 127

SARS-CoV-2     IKVCEFQFCNDPFLGVYYHKNNKSWMESEFRVYSSANNCTFEYVSQPFLMDLEGKQGNFKNLRE-----------FVFKNIDGYFKIYSK---------HTPI 210
SARS           IRACNFELCDNPFFAV--SKPMGTQTHTM--IFDNAFNCTFEYISDAFSLDVSEKSGNFKHLRE-----------FVFKNKDGFLYVYKG---------YQPI 203
MERS-CoV       LLRAFYCILE-PRSGN--HCPAGNSYTSFATYHTPATDC------SDGNYNRNASLNSFKEYFN------LRNCTFMYTYNITEDEILEWFGITQTAQGVHL 265
HCoV OC43      VSVCQYNMCEYPQTIC--HPNLGNHRKELWHLDTGVVSC---LYKRNFTYDVNADYLYFHFYQE-----------------GGTFYAYFT---------- 228
HCoV NL63      LYQPLRLTCLWPVPGL--KSSTGFVY---FNATGSDVNC------NGYQHNSVVDVMRYNLNFSANSLDNLKSGVIVFKTLQYDVLFYCS--------NSSSG 334
HCoV HKU1      ITACQYTMCEYPHTVC---KSKGSIRNESWHIDSSEPLC---LFKKNFTYNVSADWLYFHFYQE-----------------RGVFYAYYA---------- 214
HCoV 229E      SFQPLLLNCLWSVSGL--RFTTGFVY---FN-GTGRGDC------KGFSSDVLSDVIRYNLNFE----ENLRRGTILFKTSYGVVVFYCT--------NNTLV 151
BatCoV-RaTG13  IKVCEFQFCNDPFLGVYYHKNNKSWMESEFRVYSSANNCTFEYVSQPFLMDLEGKQGNFKNLRE-----------FVFKNIDGYFKIYSK---------HTPI 210

SARS-CoV-2     NLVRDLPQG--FSALEPLVDLPIGINITRFQT--LLALHRSYLTPGDSSSGWTAGAAAYY-VGYLQPRTFLLKYNENGTITDAVDCALDPLSETKCTLKS 305
SARS           DVVRDLPSG--FNTLKPIFKLPLGINITNFRA---------ILTAFSPAQDI--WGTSAAAYF-VGYLKPTTFMLKYDENGTITDAVDCSQNPLAELKCSVKS 292
MERS-CoV       FSSRYVDLY--GGNMFQFATLPVYDTIKYYSI--IPHSIRSIQSDR-KA--W------AAFY-VYKLQPLTFLLDFSVDGYIRRAIDCGFNDLSQLHCSYES 353
HCoV OC43      --DTGVVTK--F-----LFNVYLGMALSHYYV--MPLTCNSKLT-------------------LEYW-VTPLTSRQYLLAFNQDGIIFNAEDCMSDFMSEIKCKTQS 304
HCoV NL63      VLDTTIPFGPSSQPYYCFINSTINTTHVSTFVGILPPTVREIVVARTGQ--FYINGFKYFDLGFIEAVNFNVTTASA---TDFWTVAFATFVDVLVNVSA 429
HCoV HKU1      --DVGMPTT--F-----LFSLYLGTILSHYYV--MPLTCKAISSNTDNE----------TLEYW-VTPLSRRQYLLNFDEHGVITNAVDCSSSFLSEIQCKTQS 296
HCoV 229E      SGDAHIPFGTVLGNFYCFVNTTIGNETTSAFVGALPKTVREFVISRTGH--FYINGYRYFTLGNVEAVNFNVTTAET---TDFCTVALASYADVLVNVSQ 246
BatCoV-RaTG13  NLVRDLPPG--FSALEPLVDLPIGINITRFQT--LLALHRSYLTPGDSSSGWTAGAAAYY-VGYLQPRTFLLKYNENGTITDAVDCALDPLSETKCTLKS 305

SARS-CoV-2     FTVEKGIYQTSNFRVQPTESIV-RFPNITNLCPFGEVFNATRFASVYAWNRKRISNCVADYSVLYNSASFSTFKCYGVSPTKLNDLCFTNVYADSFVIRG 404
SARS           FEIDKGIYQTSNFRVVPSGDVV-RFPNITNLCPFGEVFNATKFPSVYAWERKKISNCVADYSVLYNSTFFSTFKCYGVSATKLNDLCFSNVYADSFVVKG 391
MERS-CoV       FDVESGVYSVSSFEAKPSGSVVEQAEGVE--CDFSPLLSGTP-PQVYNFKRLVFTNCNYNLTKLLSLFSVNDFTCSQISPAAIASNCYSSLILDYFSYPL 450
HCoV OC43      IAPPTGVYELNGYTVQPIADVYRRKPNLPN-CNIEAWLNDKSVPSPLNWERKTFSNCNFNMSSLMSFIQADSFTCNNIDAAKIYGMCFSSITIDKFAIPN 403
HCoV NL63      TNIQNLLYCDSPFEKLQCEHLQ---FGLQDGFYSANFLDDNVLPETYVALPIYYQHTDINFTATASF----GGSCYVCKPHQV------NISLNG----- 511
HCoV HKU1      FAPNTGVYDLSGFTVKPVATVYRRIPNLPD-CDIDNWLNNVSVPSPLNWERRIFSNCNFNLSTLLRLVHVDSFSCNNLDKSKIFGSCFNSITVDKFAIPN 395
HCoV 229E      TSIANIIYCNSVINRLRCDQLS---FDVPDGFYSTSPIQSVELPVSIVSLPVYHKHTFIVLYVDFKP-QSGGGKCFNCYPAGV------NITLANF---N 333
BatCoV-RaTG13  FTVEKGIYQTSNFRVQPTDSIV-RFPNITNLCPFGEVFNATTFASVYAWNRKRISNCVADYSVLYNSTSFSTFKCYGVSPTKLNDLCFTNVYADSFVITG 404

SARS-CoV-2     DEVRQIAPGQTGKIADYNYKLPDDFTGCVIAWNSNNLDSKVGGNYN------YLYRLFR------KSNLKPFERDISTEIYQAGST--PC---------- 480
SARS           DDVRQIAPGQTGVIADYNYKLPDDFMGCVLAWNTRNIDATSTGNYN------YKYRYLR------HGKLRPFERDISNVPFSPDGK--PC---------- 467
MERS-CoV       SMKSDLSVSSAGPISQFNYKQSFSNPTCLILATVPHNLTTITKPLK------YSY-------------INKCSRFLSDDRTEVPQLV------------ 518
HCoV OC43      GRKVDLQLGNLGYLQSFNYRIDTTATSCQLYYNLPAANVSVSRFNPSTWNKRFGFIEDSVFKPRPAGVLTNHDVVYAQHCFKAPKNFCPC---KLNGSCV 500
HCoV NL63      --NTSVCVRTSHFSIRYIYNRVKSGSPGDSSWHIYLKSGTC----P------FSFSK-----------LNNFQK-FKTICFSTVEVPGSC---------- 577
HCoV HKU1      RRRDDLQLGSSGFLQSSNYKIDISSSSCQLYYSLPLVNVTINNFNPSSWNRRYGFGSFN---------------VSSYDVVYSDHCFSVNSDFCPCADPSVVNSCV 486
HCoV 229E      ETKGPLCVDTSHFTTKYVAVYANVG-----RWSASINTGNC----P------FSFGK-----------VNNFVK-FGSVCFSLKDIPGGC---------- 396
BatCoV-RaTG13  DEVRQIAPGQTGKIADYNYKLPDDFTGCVIAWNSKHIDAKEGGNFN------YLYRLFR------KANLKPFERDISTEIYQAGSK--PC---------- 480

SARS-CoV-2     -------------------NGVEGFNC------------YF-----------PLQSYGFQPT--------------------------------------NGVGYQPYRVVVLSFE------ 516
SARS           --------------------TPPALNC------------YW-----------PLNDYGFYTT--------------------------------------TGIGYQPYRVVVLSFE------ 502
MERS-CoV       ---------------NANQYSPC-------------VSIVPSTVWEDGDYYRKQLSPLEGGGWLVASG-------------STVAMTEQLQMGFGIT---- 574
HCoV OC43      GSGPGKNNGIGTCPAGTNYLTCD------------NLCTPDPITFTGTYKCPQTKSLVGIGEHCSGLAVKSDYCGG------NSCTCRPQAFLGWSADSCLQ 584
HCoV NL63      -------------------NFPLEAT------------------------------WHYTSYTI--------------------------------------------VGALYVTWSEG------ 603
HCoV HKU1      KSKPLS------AICPAGTKYRHCDLDTTLYVNNWCRCSCLPDPISTYSPNTCPQKKVVVGIGEHCPGLGINEEKCGTQLNHSSCSCSPDAFLGWSFDSCIS 582
HCoV 229E      -------------------AMPIVAN------------------------------WAYSKYYT--------------------------------------------IGSLYVSWSDG------ 422
BatCoV-RaTG13  -------------------NGQTGLNC------------YY-----------PLYRYGFYPT--------------------------------------DGVGHQPYRVVVLSFE------ 516

SARS-CoV-2     ----------LLHA--PATVCGPKK---STNLV--KNKCVNFNFNGLTGTGVLTESNKKFL-PFQQFGRDIADTTDAVRDPQTLEILDITPCSFGGVSVI 598
SARS           ----------LLNA--PATVCGPKL---STDLI--KNQCVNFNFNGLTGTGVLTPSSKRFQ-PFQQFGRDVSDFTDSVRDPKTSEILDISPCSFGGVSVI 584
MERS-CoV       ----------VQYGTDTNSVCPKLEFANDTKIASQLGNCVEYSLYGVSGRGVFQNCTAVGV-RQQRFVYDAYQNLVGYYS-DDGNYYCLRACVSVPVSVI 662
HCoV OC43      GDKCNIFANFILHDVNSGLTCSTDLQKANTDII--LGVCVNYDLYGILGQGIFVEVNATYYNSWQNLLYDSNGNLYGFRDYIINRTFMIRSCYSGRVSAA 682
HCoV NL63      ------------NSI-TGVPYPVSGIREFSNLV--LNNCTKYNIYDYVGTGIIRSSNQSLAGGITYV--SNSGNLLGFKNVSTGNIFIVTPCNQPDQVAV 686
HCoV HKU1      NNRCNIFSNFIFNGINSGTTCSNDLLYSNTEVS--TGVCVNYDLYGITGQGIFKEVSAAYYNNWQNLLYDSNGNIIGFKDFLTNKTYTILPCYSGRVSAA 680
HCoV 229E      ------------DGI-TGVPQPVEGVSSFMNVT--LDKCTKYNIYDVSGVGVIRVSNDTFLNGITYT--STSGNLLGFKDVTKGTIYSITPCNPPDQLVV 505
BatCoV-RaTG13  ----------LLNA--PATVCGPKK---STNLV--KNKCVNFNFNGLTGTGVLTESNKKFL-PFQQFGRDIADTTDAVRDPQTLEILDITPCSFGGVSVI 598

SARS-CoV-2     TPGTNTSNQVAVLYQDVNCTEVPVAIHA---------DQLTPTWRVYSTGSNVFQTRAGCLIGAEHVNNS--YECDIPIGAGICASYQT-QTNSPRRARSVAS 689
SARS           TPGTNASSEVAVLYQDVNCTDVSTAIHA------DQLTPAWRIYSTGNNVFQTQAGCLIGAEHVDTS--YECDIPIGAGICASYHTVSLLR--------STSQ 671
MERS-CoV       Y--DKETKTHATLFGSVACEHISSTMSQYSRSTRSMLKRRDSTY------GPLQTPVGCVLGLVNSSLF-VEDCKLPLGQSLCALPDTPSTLTPRSVRSVPG 755
HCoV OC43      F--HANSSEPALLFRNIKCNYVFN----------NSLTRQLQPI------NYFDSYLGCVVNAYNSTAISVQTCDLTVGSGYCVDYS--KNRRSRGAITTGY 764
HCoV NL63      Y----QQSIIGAMTAVNESRYGLQ----------NLLQLPNFYY-----VSNGGNNCTTAVMTYSNF-----------GICADGSL-IPVRPRN------ 749
HCoV HKU1      F--YQNSSSPALLYRNLKCSYVL-----------NNISFISQPF--------YFDSYLGCVLNAVNLTSYSVSSCDLRMGSGFCIDYALPSSRRKRRGISSPY 762
```

```
HCoV 229E      Y----QQAVVGAMLSENFTSYGFS----------NVVELPKFFY-----ASNGTYNCTDAVLTYSSF-----------GVCADGSI-IAVQPRN------ 568
BatCoV-RaTG13  TPGTNASNQVAVLYQDVNCTEVPVAIHA------DQLTPTWRVYSTGSNVFQTRAGCLIGAEHVNNS--YECDIPIGAGICASYQTQTNSR--------SVAS 685

SARS-CoV-2     QSIIAYTMSLGAENSVAYSNNS---IAIPTNFTISVTTEILPVSMTKTSVDCTMYICGDSTECSNLLLQYGSFCTQLNRALTGIAVEQDKNTQEVFAQVK 786
SARS           KSIVAYTMSLGADSSIAYSNNT---IAIPTNFSISITTEVMPVSMAKTSVDCNMYICGDSTECANLLLQYGSFCTQLNRALSGIAAEQDRNTREVFAQVK 768

MERS-CoV       EMRLAS-IAFNHPIQVDQLNSSYFKLSIPTNFSFGVTQEYIQTTIQKVTVDCKQYVCNGFQKCEQLLREYGQFCSKINQALHGANLRQDDSVRNLFASVK 854
HCoV OC43      RFTNFEPFTVNSVNDSLEPVGGLYEIQIPSEFTIGNMVEFIQTSSPKVTIDCAAFVCGDYAACKSQLVEYGSFCDNINAILTEVNELLDTTQLQVANSLM 864
HCoV NL63      -------SSDNGISAIITAN--------LSIPSNWTTSVQVEYLQITSTPIVVDCATYVCNGNPRCKNLLKQYTSACKTIEDALRLSAHLETNDVSSMLTFDS 837
HCoV HKU1      RFVTFEPFNVSFVNDSVETVGGLFEIQIPTNFTIAGHEEFIQTSSPKVTIDCSAFVCSNYAACHDLLSEYGTFCDNINSILNEVNDLLDITQLQVANALM 862
HCoV 229E      -------VSYDSVSAIVTAN--------LSIPSNWTTSVQVEYLQITSTPIVVDCSTYVCNGNVRCVELLKQYTSACKTIEDALRNSARLESADVSEMLTFDK 656
BatCoV-RaTG13  QSIIAYTMSLGAENSVAYSNNS---IAIPTNFTISVTTEILPVSMTKTSVDCTMYICGDSTECSNLLLQYGSFCTQLNRALTGIAVEQDKNTQEVFAQVK 782

SARS-CoV-2     QIYKTPPIKDFG-----G-FNFSQILPD-PSK---PSKRSFIEDLLFNKVTLADAGFIKQ-YGDCL--GDIAARDLICAQKFNGLTVLPPLLTDEMIAQY 873
SARS           QMYKTPTLKYFG-----G-FNFSQILPD-PLK---PTKRSFIEDLLFNKVTLADAGFMKQ-YGECL--GDINARDLICAQKFNGLTVLPPLLTDDMIAAY 855
MERS-CoV       SSQSSPIIPGFG--------GDFNLTLLEPVSISTGS-RSARSAIEDLLFDKVTIADPGYMQG-YDDCMQQGPASARDLICAQYVAGYKVLPPLMDVNMEAAY 947
HCoV OC43      NGVTLSTKLKDGVNFNVDDINFSPVLGCLGSECSKASSRSAIEDLLFDKVKLSDVGFVEA-YNNCT--GGAEIRDLICVQSYKGIKVLPPLLSENQISGY 961
HCoV NL63      NAFSLANVTSFG--------D-YNLSSVLPQRNIRSSRIAGRSALEDLLFSKVVTSGLGTVDVDYKSCT--KGLSIADLACAQYYNGIMVLPGVADAERMAMY 929
HCoV HKU1      QGVTLSSNLNTNLHSDVDNIDFKSLLGCLGSQCG-SSSRSLLEDLLFNKVKLSDVGFVEA-YNNCT--GGSEIRDLLCVQSFNGIKVLPPILSETQISGY 958
HCoV 229E      KAFTLANVSSFG--------D-YNLSSVIPSLPTSGSRVAGRSAIEDILFSKLVTSGLGTVDADYKKCT--KGLSIADLACAQYYNGIMVLPGVADAERMAMY 748
BatCoV-RaTG13  QIYKTPPIKDFG-----G-FNFSQILPD-PSK---PSKRSFIEDLLFNKVTLADAGFIKQ-YGDCL--GDIAARDLICAQKFNGLTVLPPLLTDEMIAQY 869

SARS-CoV-2     TSALLAGTITSGWTFGAGAALQIPFAMQMAYRFNGIGVTQNVLYENQKLIANQFNSAIGKIQDSLSSTA----------------------SALGKLQDVVNQNAQAL 959
SARS           TAALVSGTATAGWTFGAGAALQIPFAMQMAYRFNGIGVTQNVLYENQKQIANQFNKAISQIQESLTTTS----------------------TALGKLQDVVNQNAQAL 941
MERS-CoV       TSSLLGSIAGVGWTAGLSSFAAIPFAQSIFYRLNGVGITQQVLSENQKLIANKFNQALGAMQTGFTTTN----------------------EAFHKVQDAVNNNAQAL 1033
HCoV OC43      TLAATSASLFPPWTAA----AGVPFYLNVQYRINGLGVTMDVLSQNQKLIANAFNNALYAIQEGFDATN----------------------SALVKIQAVVNANAEAL 1043
HCoV NL63      TGSLIGGMVLGGLTSA------AAIPFSLALQARLNYVALQTDVLQENQKILAASFNKAINNIVASFSSVNDAITQTAEAIHTVTIALNKIQDVVNQQGSAL 1025
HCoV HKU1      TTAATVAAMFPPWSAA----AGVPFSLNVQYRINGLGVTMDVLNKNQKLIANAFNKALLSIQNGFTATN----------------------SALAKIQSVVNANAQAL 1040
HCoV 229E      TGSLIGGIALGGLTSA------VSIPFSLAIQARLNYVALQTDVLQENQKILAASFNKAMTNIVDAFTGVNDAITQTSQALQTVATALNKIQDVVNQQGNSL 844
BatCoV-RaTG13  TSALLAGTITSGWTFGAGAALQIPFAMQMAYRFNGIGVTQNVLYENQKLIANQFNSAIGKIQDSLSSTA----------------------SALGKLQDVVNQNAQAL 955

SARS-CoV-2     NTLVKQLSSNFGAISSVLNDILSRLDKVEAEVQIDRLITGRLQSLQTYVTQQLIRAAEIRASANLAATKMSECVLGQSKRVDFCGKGYHLMSFPQSAPHG 1059
SARS           NTLVKQLSSNFGAISSVLNDILSRLDKVEAEVQIDRLITGRLQSLQTYVTQQLIRAAEIRASANLAATKMSECVLGQSKRVDFCGKGYHLMSFPQAAPHG 1041
MERS-CoV       SKLASELSNTFGAISASIGDIIQRLDVLEQDAQIDRLINGRLTTLNAFVAQQLVRSESAALSAQLAKDKVNECVKAQSKRSGFCGQGTHIVSFVVNAPNG 1133
HCoV OC43      NNLLQQLSNRFGAISASLQEILSRLDALEAEAQIDRLINGRLTALNAYVSQQLSDSTLVKFSAAQAMEKVNECVKSQSSRINFCGNGNHIISLVQNAPYG 1143
HCoV NL63      NHLTSQLRHNFQAISNSIQAIYDRLDSIQADQQVDRLITGRLAALNAFVSQVLNKYTEVRGSRRLAQQKINECVKSQSNRYGFCGNGTHIFSIVNSAPDG 1125
HCoV HKU1      NSLLQQLFNKFGAISSSLQEILSRLDNLEAQVQIDRLINGRLTALNAYVSQQLSDITLIKAGASRAIEKVNECVKSQSPRINFCGNGNHILSLVQNAPYG 1140
HCoV 229E      NHLTSQLRQNFQAISSSIQAIYDRLDTIQADQQVDRLITGRLAALNVFVSHTLTKYTEVRASRQLAQQKVNECVKSQSKRYGFCGNGTHIFSIVNAAPEG 944
BatCoV-RaTG13  NTLVKQLSSNFGAISSVLNDILSRLDKVEAEVQIDRLITGRLQSLQTYVTQQLIRAAEIRASANLAATKMSECVLGQSKRVDFCGKGYHLMSFPQSAPHG 1055

SARS-CoV-2     VVFLHVTYVPAQEKNFTTAPAICHDG---KAHFPREGVFV--SNGTH-------WFVTQRNFYEPQIITTDNTFVSGNCDVVIGIVNNTVYDPLQPEL--DS 1147
SARS           VVFLHVTYVPSQERNFTTAPAICHEG---KAYFPREGVFV--FNGTS-------WFITQRNFFSPQIITTDNTFVSGNCDVVIGIINNTVYDPLQPEL--DS 1129
MERS-CoV       LYFMHVGYYPSNHIEVVSAYGLCDAANPTNCIAPVNGYFI--KTNNTRIVDEWSYTGSSFYAPEPITSLNTKYVAP-QVTYQNISTNLPPPLLGNSTGID 1230
HCoV OC43      LYFIHFSYVPTKYVTARVSPGLCIAGD--RGIAPKSGYFV--NVNNT-------WMYTGSGYYYPEPITENNVVVMSTCAVNYTKAPYVMLNTSIPNL--PD 1232
HCoV NL63      LLFLHTVLLPTDYKNVKAWSGICVDGI--YGYVLRQPNLVLYSDNGV-------FRVTSRVMFQPRLPVLSDFVQIYNCNVTFVNISRVELHTVIPDY--VD 1216
HCoV HKU1      LLFIHFSYKPTSFKTVLVSPGLCLSGD--RGIAPKQGYFI--KQNDS-------WMFTGSSYYYPEPISDKNVVFMNSCSVNFTKAPFIYLNNSIPNL--SD 1229
HCoV 229E      LVFLHTVLLPTQYKDVEAWSGLCVDGT--NGYVLRQPNLALYKEGNY-------YRITSRIMFEPRIPTMADFVQIENCNVTFVNISRSELQTIVPEY--ID 1035
BatCoV-RaTG13  VVFLHVTYVPAQEKNFTTAPAICHDG---KAHFPREGVFV--SNGTH-------WFVTQRNFYEPQIITTDNTFVSGSCDVVIGIVNNTVYDPLQPEL--DS 1143

SARS-CoV-2     FKEELDKY-FKNHTSPDVDLGDISGINASVVNIQKEID----------------------RLNEVAKNLNESLIDLQELGKYEQYIKWPWYIWLGFIAGLIAIVMVTI 1232
SARS           FKEELDKY-FKNHTSPDVDLGDISGINASVVNIQKEID----------------------RLNEVAKNLNESLIDLQELGKYEQYIKWPWYVWLGFIAGLIAIVMVTI 1214
MERS-CoV       FQDELDEF-FKNVSTSIPNFGSLTQINTTLLDLTYEML----------------------SLQQVVKALNESYIDLKELGNYTYYNKWPWYIWLGFIAGLVALALCVF 1315
HCoV OC43      FKEELDQW-FKNQTSVAPDL-SLDYINVTFLDLQVEMN----------------------RLQEAIKVLNQSYINLKDIGTYEYYVKWPWYVWLLICLAGVAMLVLLF 1316
HCoV NL63      VNKTLQEFAQNLPKYVKPNF-DLTPFNLTYLNLSSELKQLEAKTASLFQTTVELQGLIDQINSTYVDLKLLNRFENYIKWPWWVWLIISVVFVVLLSLLV 1315
HCoV HKU1      FEAEFSLW-FKNHTSIAPNLTFNSHINATFLD---------------------------------------------------------------- 1260
HCoV 229E      VNKTLQELSYKLPNYTVPDL-VVEQYNQTILNLTSEISTLENKSAELNYTVQKLQTLIDNINSTLVDLKWLNRVETYIKWPWWVWLCISVVLIFVVSMLL 1134
BatCoV-RaTG13  FKEELDKY-FKNHTSPDVDLGDISGINASVVNIQKEID----------------------RLNEVAKNLNESLIDLQELGKYEQYIKWPWYIWLGFIAGLIAIIMVTI 1228

SARS-CoV-2     MLCCMTSCCSCLKGCCSCGSCCKFDEDDSEPVLKGVKLHYT 1273
SARS           LLCCMTSCCSCLKGACSCGSCCKFDEDDSEPVLKGVKLHYT 1255
MERS-CoV       FILCCTGCGTNCMGKLKCNRCC--DRYEEYD-LEPHKVHVH 1353
HCoV OC43      FICCCTGCGT--SCFKKCGGCC--DDYTGYQELVIKTSHDD 1353
HCoV NL63      FCCLSTGCCGCCNCLTSSMRGCCDCGSTKLPYYEFEKVHVQ 1356
HCoV HKU1      ----------------------------------------- 1260
HCoV 229E      LCCCSTGCCGFFSCFASSIRGC--CESTKLPYYDVEKIHIQ 1173
BatCoV-RaTG13  MLCCMTSCCSCLKGCCSCGSCCKFDEDDSEPVLKGVKLHYT 1269
```

Figure S2. Multiple sequence alignment of Sars-CoV-2 Spike protein [P0DTC2] and endemic coronaviruses. Identified cross-reactive sites (yellow), correspondent identical residues (red) in coronaviruses (Sars-CoV-1 [P59594], MERS-CoV [K9N5Q8], HCoV-OC43 [P36334], HCoV-NL63 [Q6Q1S2], HCoV-HKU1 [Q14EB0], HCoV-229E [P15423], BatCoV-RaTG13 [A0A6B9WHD3]), similar amino acids (gray) were highlighted. Analysis was performed using MEGA11 software, and alignment was performed using the MUSCLE algorithm.

Table S2. Information of serum samples used in the study and dose regimen for COVID-19 vaccines.

Sample	Gender	Dose regimen	Days after vaccination
**M3**	F	A	70
**M4**	F	A	50
**M5**	M	A	97
**M7**	F	A	17

**M9**	M	A	24
**M10**	M	A	11
**M11**	M	A	46
**M12**	F	A	92
**M13**	M	A	91
**M14**	F	A	90
**M17**	M	A	89
**M20**	M	A	88
**M23**	M	A	60
**M24**	F	A	54
**M27**	F	A	39
**M28**	F	A	37
**M29**	F	A	17
**M30**	F	A	80
**M34**	F	A	40
**M37**	F	A	37
**M39**	F	A	16
**M40**	F	A	27
**M43**	F	A	35
**M46**	F	A	33
**B1**	M	A/A/A/P	236
**B2**	F	A/A/P/P	259
**B3**	M	A/A/P/A	142
**B4**	M	A/A/P/P	122
**B5**	F	A/A/P/J	303
**B6**	F	A/A/P	401
**B7**	F	A/A/P/A	261

*A= Astrazeneca viral vector vaccine; P = Pfizer Spike mRNA vaccine; J = Janssen virus-based technology vaccine

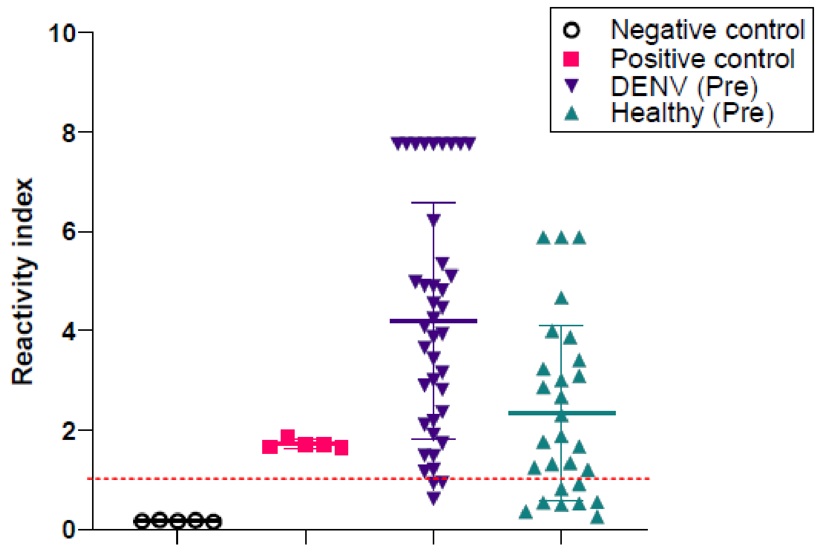

Figure S3. Analysis of ELISA test for DENV (Serotypes 1-4). Reactivity index of serum samples from healthy (n=28) and DENV (n=40) pre-pandemic individuals.
